# Accuracy of Calf Circumference Measurement, SARC-F Questionnaire, and Ishii's Score for Screening Stroke-Related Sarcopenia

**DOI:** 10.3389/fneur.2022.880907

**Published:** 2022-04-29

**Authors:** Ruihong Yao, Liqing Yao, Changli Yuan, Bu-Lang Gao

**Affiliations:** ^1^Medical Imaging Department, First Affiliated Hospital of Kunming Medical University, Kunming, China; ^2^Rehabilitation Medicine Department, Second Affiliated Hospital of Kunming Medical University, Kunming, China

**Keywords:** stroke, sarcopenia, accuracy, screening, sensitivity

## Abstract

**Objective:**

The purpose of this study was to investigate the accuracy of sarcopenia diagnosis in patients with stroke using calf circumference (CC), SARC-F questionnaire, and Ishii's score in comparison with the Asian Working Group for Sarcopenia 2019 (AWGS) sarcopenia diagnostic criteria.

**Materials and Methods:**

In this cross-sectional study, a total of 364 consecutive patients with stroke were enrolled and evaluated with the CC measurement, SARC-F questionnaire, and Ishii's score. The diagnostic accuracy was analyzed.

**Results:**

Based on the AWGS criteria, sarcopenia was present in 180 (49.5%) patients, with an age range of 49–74 (mean 63 ± 14.7) years. In all patients, the cutoff value of CC in the accuracy of diagnosing sarcopenia was 30.5 cm, with an AUC of 0.85, sensitivity of 81.8%, specificity of 90.1%, Kappa value of 0.72, and Youden index of 0.72. In the accuracy of diagnosing sarcopenia in all patients, Ishii's score had a cutoff value of 118, AUC of 0.78, sensitivity of 90.1%, specificity of 36.0%, Kappa value of 0.4, and Youden index of 0.55. For accuracy of diagnosing sarcopenia, the SARC-F questionnaire had a cutoff value of 5, AUC of 0.731, sensitivity of 94.7%, specificity of 40%, Kappa value of 0.34, and Youden index of 0.41.

**Conclusions:**

Based on the AWGS criteria, calf circumference measurement has the optimal performance in screening stroke-related sarcopenia compared with the SARC-F questionnaire and Ishii's score. In patients with stroke, the cutoff value of calf circumference for sarcopenia is < 31 cm in men and 30 cm in women, and with an AUC of 0.85.

## Introduction

Stroke is the first leading cause of death globally, with approximately 2,500,000 strokes every year in China, and 80% of patients with stroke are disabled (1). With high morbidity, mortality, and recurrence rates, stroke mainly occurs in the elderly over 60 years (2). Sarcopenia refers to the reduction in muscle quantity and mass accompanied by the decline of muscle strength and body function mainly in elderly people over the age of 60 years (3). The incidence of sarcopenia is approximately 20% in people between 60 and 70 years of age but increases greatly to 50% in people over the age of 80 years (4–6). Stroke results in the loss of limb motor neuron function, muscle inactivity, and eventually muscle atrophy and weakness (7–9). Loss of skeletal muscle mass and strength in patients with stroke is referred to as stroke-related sarcopenia (SRS) and is a common complication, affecting approximately 56% of all patients suffering from subacute stroke (10, 11). Following SRS, an increased risk for malnutrition will ensue. In fact, this threat has been quantified to be a 4-fold increase for individuals with stroke compared to patients with stroke but without sarcopenia (12). This is a vicious cycle, probably resulting in longer hospital stays and recovery, higher healthcare costs, more complications, and increased rates of morbidity and mortality compared to those without sarcopenia (13). Earlier identification of sarcopenia will be helpful in reducing the risk of malnutrition, and earlier intervention for sarcopenia had been demonstrated to result in less disability, decreased duration of care, and reduced mortality at 1 year (14). Therefore, early identification of SRS is critical to the prevention of these complications, initiation of earlier intervention, and acceleration of recovery (15).

There was no gold standard for the diagnosis of sarcopenia. Different sarcopenia consensuses may have different sarcopenia diagnosis approaches and criteria. For the European Working Group on Sarcopenia in Older People updated diagnosis 2018 (EWGSOP2), first, the gait speed was measured, and if it was < 0.8 m/s, the skeletal muscle mass was measured. When the gait speed was >0.8 m/s, handgrip strength was measured, and if the handgrip strength was normal, sarcopenia was excluded. If the handgrip strength was decreased below the normal value, the skeletal muscle mass was measured for diagnosis of sarcopenia. The appendicular muscle mass index (ASMI) for diagnosis of sarcopenia was <7.0 kg/m^2^ in men and <5.5 kg/m^2^ in women for EWGSOP (16). For the International Working Group on Sarcopenia 2011 (IWGS), the ASMI for diagnosis of sarcopenia was ≤7.23 kg/m^2^ in men and ≤5.67 kg/m^2^ in women (17). Because of the differences in region and race in China, the Asian Work Group for Sarcopenia (AWGS) 2019 criteria were primarily used, measuring both appendicular muscle mass and muscle strength. The skeletal muscle mass index (SMI) was calculated from measured appendicular muscle mass divided by height squared (m^2^). The cutoff value for SMI was <7.0 kg/m^2^ and <5.7 kg/m^2^ by the bioelectrical impedance analysis (BIA) examination for men and women, respectively. Handgrip strength was measured on the non-paretic side of patients with paralysis, using a digital hand dynamometer in the standing or seated position, and low muscle strength was defined as <28 kg and <18 kg for men and women, respectively (18). Sarcopenia was diagnosed when both SMI and muscle strength were less than the normal values.

Currently, the evaluation of sarcopenia can be performed through several screening tools. SARC-F is a questionnaire that rapidly screens sarcopenia using self-reported data about falls, stair climbing, rise from a chair, assistance walking, and strength (19). SARC-F is recommended by AWGS 2019 as the first tool with very good specificity to screen sarcopenia in community older people. Ishii's score is another rapid screening tool for sarcopenia by measuring handgrip strength and calf circumference. It was also mainly used in the community older people in Japan and other countries (20). Calf circumference (CC) was used as a case-finding tool to diagnose sarcopenia. Some studies have reported the screening ability of CC to identify sarcopenia in community-dwelling older adults and older patients with stroke (21, 22). These findings suggest that CC might be a useful tool for the case finding of sarcopenia. In clinics, the prevalence of patients with stroke with sarcopenia is high. Muscle mass loss in patients with stroke was mainly caused by paralysis-related inactivity and dysphagia-related malnutrition. Skeletal muscle atrophy occurs first on the paralyzed side followed by the non-paralyzed side. Approximately half of the patients with stroke suffer from sarcopenia that may extend the duration of hospitalization. It is currently unknown the predictive ability of CC, SARC-F, and Ishii's score for the diagnosis of sarcopenia in patients with stroke, and their diagnostic accuracy and cutoff values in diagnosing sarcopenia in patients with stroke compared to community-dwelling older adults. Ease of use of both tools may facilitate rapid and extensive screening of SRS. Although these tools have been used in assessing sarcopenia in other races and nations, they have not been widely used in China, especially in evaluating SRS. Some studies have compared the accuracy of the two methods (23), with the optimal sensitivity and largest area under the curve (AUC) in the receiver operating curve (ROC) analysis to identify sarcopenia in older people demonstrated in the Ishii's formula as compared to five other sarcopenia diagnostic definitions, including SARC-F (20). Although these screening tools could predict the prevalence of adverse outcomes such as falls, fractures, and death (24), their prognostic power remains unknown. It was hypothesized that the calf circumference measurement, SARC-F, and Ishii's score could all be used to evaluate SRS in patients with stroke, and this study was consequently performed to test the accuracy of calf circumference measurement, SARC-F, and Ishii's scores in the screening of SRS in patients with stroke.

## Materials and Methods

This cross-sectional study was approved by the ethics committees of the Second Affiliated Hospital of Kunming Medical University. Written informed consent was acquired from each patient or their family members to participate. Consecutive patients with suspected SRS between May 2010 and July 2021 were enrolled. The inclusion criteria were the patients with stroke for the first time, aged 18–80 years, and with a stroke duration from 2 weeks to 6 months regardless of being able to walk independently. The exclusion criteria were patients with cardiac, hepatic, renal, and pulmonary failure, severe systematic diseases, malignant tumors, mental illness, hereditary muscle diseases, osteoarthritis, the onset of a transient ischemic attack, and pregnancy. Four doctors who were blind to the clinical data conducted CC measurement, SARC-F scoring, Ishii screening, standard BIA, and handgrip strength examination. If in disagreement, a consensus was reached through discussion.

Ischemic and hemorrhagic strokes were characterized by a focal cerebral infarct or hemorrhage with neurological deficits as confirmed by diffusion-weighted magnetic resonance imaging (MRI) and computed tomography (CT) (25, 26). SRS was defined as “muscle failure due to stroke” characterized by any loss of muscle mass and decrease of muscle strength or physical function. For this study, the sarcopenia diagnosis criteria were based on the 2019 AWGS screening and diagnostic criteria, with the sarcopenia screen criteria as calf circumference (CC) < 34 cm in men and CC < 33 cm in women. Sarcopenia was diagnosed if patients met the following two to three conditions (18): (1) ASMI of limbs measured by BIA < 7.0 kg/m^2^ in men and < 5.7 kg/m^2^ in women, (2) muscle strength: the maximal grip strength < 28 kg in men and < 18 kg in women, and (3) physical performance function: walking speed ≤ 0.8 m/s for both men and women.

### Sample Size Calculation Formula

The sensitivity of CC screening for sarcopenia was 88% in men and 76% in women, with a specificity of 91% for men and 73% for women according to the 2019 AWGS (27). The sensitivity and specificity of SARC-F in comparison with AWGS 2019 as the gold standard were 25.0 and 81.4%, respectively (20). The sensitivity and specificity of Ishii's score in comparison with AWGS 2019 as the gold standard were 84.9 and 88.2%, respectively (28). According to the sensitivity and specificity, the sample size was calculated and the maximal number of patients was selected. The significance test level was α = 0.05, with the allowable error δ = 0.08. The sample size of the screening test was calculated as N = Zα^2^p (1-p)/δ^2^. The total sample size was 340 patients.

### Measurement

The clinical data were recorded, including sex, age, number of days from stroke onset to hospital admission, and stroke type. The patient's calf circumference, mid-arm circumference, handgrip strength, and BIA were measured at admission. The measurements included paralyzed and non-paralyzed calf circumference, mid-arm circumference, handgrip strength, and BIA. The non-paralyzed calf circumference was used for diagnosis screen.

The SARC-F questionnaires were filled by a single qualified doctor who did not perform other measurements. The SARC-F scores were applied to reflect the muscle strength and body function changes in patients with stroke, ranging from 0 (best) to 10 (worse) with three scores in each of five items, including strength, walking, rising from a chair, climbing 10 stairs, and falls, with the total scores ≥ 4, indicating positive screening for sarcopenia.

A trained doctor checked for pitting edema before the CC measurement and then measured the two sides, including the paretic and non-paretic CC of patients with paralysis at admission in a sitting or supine position, the knees bent, and the hips bent at 90°. The feet were naturally placed on the ground or on the bed with the feet and ankles relaxed. The tester faced the subject and determined the thickest part of the bilateral lower legs. The flexible tape ruler was used to measure the CC after being wrapped perpendicularly around the lower leg axis, with a measurement error of < 0.1 cm. Both upper limb circumferences were measured in the same way. To decrease the effect of edema, the measurements were taken in the morning. Both legs were measured three times and the highest value of the measurements was recorded. Low calf circumference was referred to <34 and <33 cm for men and women, respectively.

Handgrip strength that reflected muscle strength was measured on the non-paretic side of patients with paralysis, using the Jamar electronic grip hand dynamometer. The patient could stand, sit, or lie with the arms straight by the sides, depending on the patients' motor ability. The maximal grip strength was measured three times, with the highest measurements being recorded. Low muscle strength was referred to <28 and < 18 kg for men and women, respectively.

Ishii's score included three variables: age, grip strength, and CC. The higher the score, the higher the prevalence of sarcopenia. The gender-specific sarcopenia calculation formula was derived using the above three variables: male score = 0.62 × (age – 64)−3.09 × (grip strength – 50) - 4.64 × (CC – 42), and female score = 0.8 × (age – 64)−5.09 × (grip strength – 34)−3.28 × (CC – 42). A score greater than 105 and 120 indicated sarcopenia in men and women, respectively.

The muscle mass was measured with the bioelectrical impedance analysis (Body composition analyzer, BIA, In Body S10, Korea) in the supine position after fasting for 8 h at admission. The measurements were taken in the morning after urination or defecation. The ASMI was calculated from appendicular muscle mass divided by height squared (m^2^). The referred cutoff value for ASMI was <7.0 kg/m^2^ and <5.7 kg/m^2^ for men and women, respectively.

### Statistical Analysis

Data analyses were performed with the SPSS software (version 23, IBM, Chicago, IL, USA). If the continuous measurement data were in the normal distribution, they were presented as mean ± standard deviation and tested with the independent *t*-test. If the measurement data were in skew distribution, they were presented as median and interquartile ranges and tested with the nonparametric test. Enumeration data were presented as numbers and percentages and tested with the chi-square test. The ROC curve analysis and cutoff of continuous variables were performed according to the sensitivity and specificity of each subject, and the Youden index was calculated. The CC value corresponding to the maximal value of the Youden index was used as the best cutoff point of SRS. The sensitivity, specificity, and AUC of the ROC curve were used to evaluate the accuracy of CC measurement for SRS diagnosis. All data were analyzed hierarchically according to gender and stroke type. The significant *p* was set as <0.05.

## Results

### Subjects

**Figure 1:**
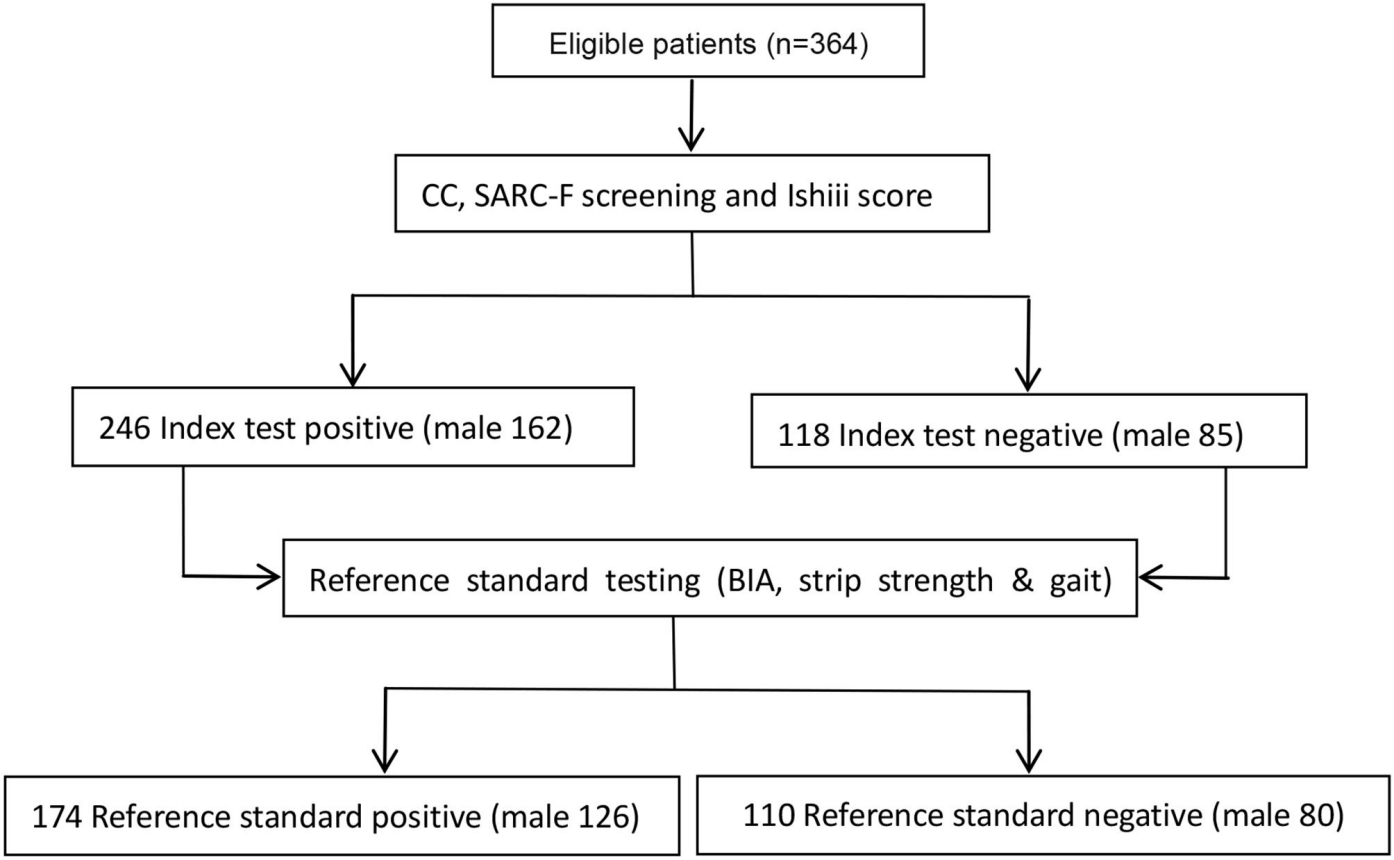
Flow of Screening for stroke-related sarcopenia (SRS) with a bioelectrical impedance analysis (BIA), strip strength, and gait testing (reference standard test).

A total of 364 participants were enrolled, including 270 (74.2%) male and 94 (25.8%) female patients, with a mean age of 57 years (48–69) and an average BMI of 22.1 kg/m^2^ (range 20.7–24.4 kg /m^2^) (Figure 1, Table 1). Ischemic stroke was presented in 189 (51.2%) patients and hemorrhagic in 175 (48.8%). No significant (*p* > 0.05) differences were found in the SARC-F scores, male number, left mid-arm circumference, right CC, left grip strength, and incidence of SARS between ischemic and hemorrhagic strokes. Patients with ischemic stroke were significantly (*p* = 0.001) older than those with hemorrhagic stroke, and the right handgrip strength was significantly (*p* = 0.001) greater in patients with ischemic stroke than those with hemorrhagic stroke. The incidence of SRS was insignificantly (*p* > 0.05) lower in patients with ischemic stroke (Table 2).

**Table 1:** Data of patients with and without sarcopenia.

	**Total**	**Sarcopenia**	**Non-sarcopenia**	***p***
No.	364	180 (49.5%)	184 (50.5%)	
Marriage	314 (86.1%)	73 (41.3%)	109 (58.7%)	0.17
F/M	94/270	46/134	48/136	0.2
Age, years	57 (48.0–69.0)	62.0 (49.0–74)	54.0 (44.0–64.0)	0.008
SARC-F	8 (6–8)	8 (8–8)	6 (4–8)	0
Height, m	1.68 (1.63–1.72)	1.68 (1.60–1.72)	1.68 (1.65–1.75)	0.32
Weight, kg	63.4 ± 10.1	58.4 ± 0.9	67.4 ± 1.1	0
Weight loss, kg	5 (3.0–8.0)	5.0 (3–8.0)	5.0 (3.0–10.0)	0.547
BMI,kg/m^2^	22.1 (20.7–24.4)	20.8 (19.3–22.3)	23.6 (21.6–25.3)	0
Recovery time, d	14 (4.0–50.0)	10.0 (3.0–30)	17.0 (6.0–54.0)	0.04
Disease course, d	51 (26–90)	45.0 (26.0–80.0)	60.0 (27.0–120.0)	0.23
left grip, kg	5 (0–17)	3.0 (0–11.0)	9.0 (0–20.0)	0
Right grip, kg	5 (0–20)	0.0 (0–8.0)	13.0 (1.0–25.0)	0
Left upper limb, cm	29 ± 3.3	27.1 ± 0.3	30.5 ± 0.3	0
Right upper limb, cm	29 ± 3.6	26.7 ± 0.33	30.8 ± 0.32	0
Left CC, cm	31.7 ± 3.5	29.2 ± 0.3	33.8 ± 0.3	0
Right CC, cm	31.6 ± 3.4	29.2 ± 0.27	33.6 ± 0.29	0
Healthy grip, kg	16 (6.8–24.0)	8.0 (3.0–18.0)	20.0 (13.0–27.0)	0
Healthy calf circumference	31.7 ± 3.5	29.2 ± 0.3	33.7 ± 0.3	0
Left upper limb mass, kg	2.4 ± 0.7	2.2 ± 0.04	2.66 ± 0.98	0
Right upper limb mass, kg	2.4 ± 0.6	2.16 ± 0.41	2.64 ± 0.07	0
Left lower limb muscle mass, kg	7.4 ± 1.6	6.40 ± 0.13	8.2 ± 0.17	0
Right lower limb muscle mass, kg	7.3 ± 1.9	6.37 ± 0.13	8.1 ± 0.2	0
Trunk muscle mass, kg	20.9 ± 4.1	19.3 ± 3.5	22.4 ± 3.2	0
Basal metabolic calories, kal	1359 ± 189	1259 ± 13.75	1438 ± 21.5	0
ASM	6.9	6.2(5.5–6.6)	7.5(7.1–8.1)	0

**Table 2:** Diagnostic accuracy of three screening tools for patients with ischemic or hemorrhagic stroke.

	**Ischemic stroke**	**Hemorrhagic stroke**	***p***
Total	189	175	
SARC-F	6.9	7.9	0.4
Age	59.1	52.3	0
Male	141	126	0.615
LMAC	29.2	29	0.74
RMAC	29.8	29	0.23
LGS	9	9.1	0.91
RGS	12.3	7.6	0.001
LCC	32.4	31.4	0.32
RCC	32.4	31.4	0.34
SRS count	70	84	
SRS percentage	37%	48%	0.078

*LMAC, left mid-arm circumference; RMAC, right mid-arm circumference; LGS, left grip strength; RGS, right grip strength; LCC, left calf circumference; RCC, right calf circumference*.

Based on the 2019 AWGS screening and diagnostic criteria of sarcopenia, sarcopenia was present in 180 (49.5%) patients, with an age range of 49–74 (mean 63 ± 14.7) years, body weight of 34–98 kg (mean 58.4 ± 0.9), and height of 1.68 (range 1.6–1.72) m, including 144 (39.6%) male patients with a median age of 61 and 36 (9.9%) female patients with a median age of 66.

The mean time from stroke onset to presentation was 67 days (3–159), the median time from hospitalization to initiation of physical treatment was 21 days (7.0–78.0), and the median time to the first walking was 17 days (ranging from 10.0 to 35.0).

### Accuracy of CC for Diagnosing Sarcopenia

In the total cohort of patients, the cutoff value of CC measurement in the accuracy of diagnosing sarcopenia was 30.5 cm, with an AUC of 0.85, sensitivity of 81.8%, specificity of 90.1%, Kappa value of 0.72, and Youden index of 0.72 (Table 3). For male patients with sarcopenia, the CC measurement had a cutoff value of 31 cm, AUC of 86.0%, sensitivity of 78.9%, specificity of 95.7%, Kappa value of 0.828, and Youden index of 0.74. For diagnosing sarcopenia in female patients, CC had a cutoff value of 30 cm, AUC of 83%, sensitivity of 84.2%, specificity of 82%, Kappa value of 0.8, and Youden index of 0.66 (Table 3). The positive and negative likelihood ratios of CC measurement for SRS were 8.34 and 0.1 for all patients, 18 and 0.27 for men, and 4.64 and 0.187 for women, respectively. The positive and negative predictive values of CC measurement were 88.9 and 83.7% for all patients, 95 and 81.5% for men, and 80.0 and 85.7% for women, respectively.

**Table 3:** Diagnostic accuracy of three screening tools.

**Variables**	**CC-screen**			**SARC-F**	**Ishii**		
	**Total**	**Male**	**Female**	**Total**	**Total**	**Male**	**Female**
Sensitivity	81.80%	78.90%	84.20%	94.70%	90.10%	94.40%	85.70%
Specificity	90.10%	95.70%	82%	40.00%	36.00%	44.40%	28.60%
PPV	88.90%	95%	80.00%	54.50%	55.00%	57%	54.00%
NPV	83.70%	81.50%	85.70%	90.90%	62.00%	57%	67.00%
PLR	8.34	18	4.64	1.6	2.3	2.5	2.1
NLR	0.1	0.27	0.187	0.06	0.05	0.059	0.041
AR	86.00%	87.10%	83%	65.00%	60.10%	66.70%	57%
AUC	0.85	0.828	0.8	0.731	0.78	0.8	0.77
Kappa	0.72	0.74	0.66	0.34	0.4	0.469	0.4
Youden	0.72	0.66	0.62	0.41	0.55	0.57	0.54
Cut-off	31.3 cm	31 cm	30 cm	5	118	118	162

*PPV, positive predictive value; NPV, negative predictive value; PLR, positive likelihood ratio; NLR, negative likelihood ratio; AR, accuracy rate; AUC, area under the curve*.

### Accuracy of Ishii's Score

In the accuracy of diagnosing sarcopenia in the total cohort of patients, Ishii's score had a cutoff value of 118, AUC of 0.78, sensitivity of 90.1%, specificity of 36.0%, Kappa value of 0.4, and Youden index of 0.55 (Table 3). For male patients, the Ishii's score had a cutoff value of 118, AUC of 66.7%, sensitivity of 94.4%, specificity of 44.4%, Kappa value of 0.8, and Youden index of 0.469. For female patients, Ishii's score had a cutoff value of 162, AUC of 57%, sensitivity of 85.7%, specificity of 28.6%, Kappa value of 0.77, and Youden index of 0.4 (Table 3). The positive and negative likelihood ratios of Ishii's score for diagnosing SRS were 82.3 and 0.05 for all patients, 2.5 and 0.059 for men, and 2.1 and 0.041 for women, respectively. The positive and negative predictive values of Ishii's score were 55 and 62% for all patients, 57 and 57% for men, and 54 and 67% for women, respectively.

### Accuracy of SARC-F Questionnaire

For the accuracy of diagnosing sarcopenia, the SARC-F questionnaire had a cutoff value of 5, AUC of 0.731, sensitivity of 94.7%, specificity of 40%, Kappa value of 0.34, and Youden index of 0.41 (Table 3). The positive and negative likelihood ratios of SARC-F for SRS were 1.6 and 0.06 for all patients, and the positive and negative predictive values were 54.5 and 90.9%, respectively.

### ROC Curve Analysis

**Figure 2:**
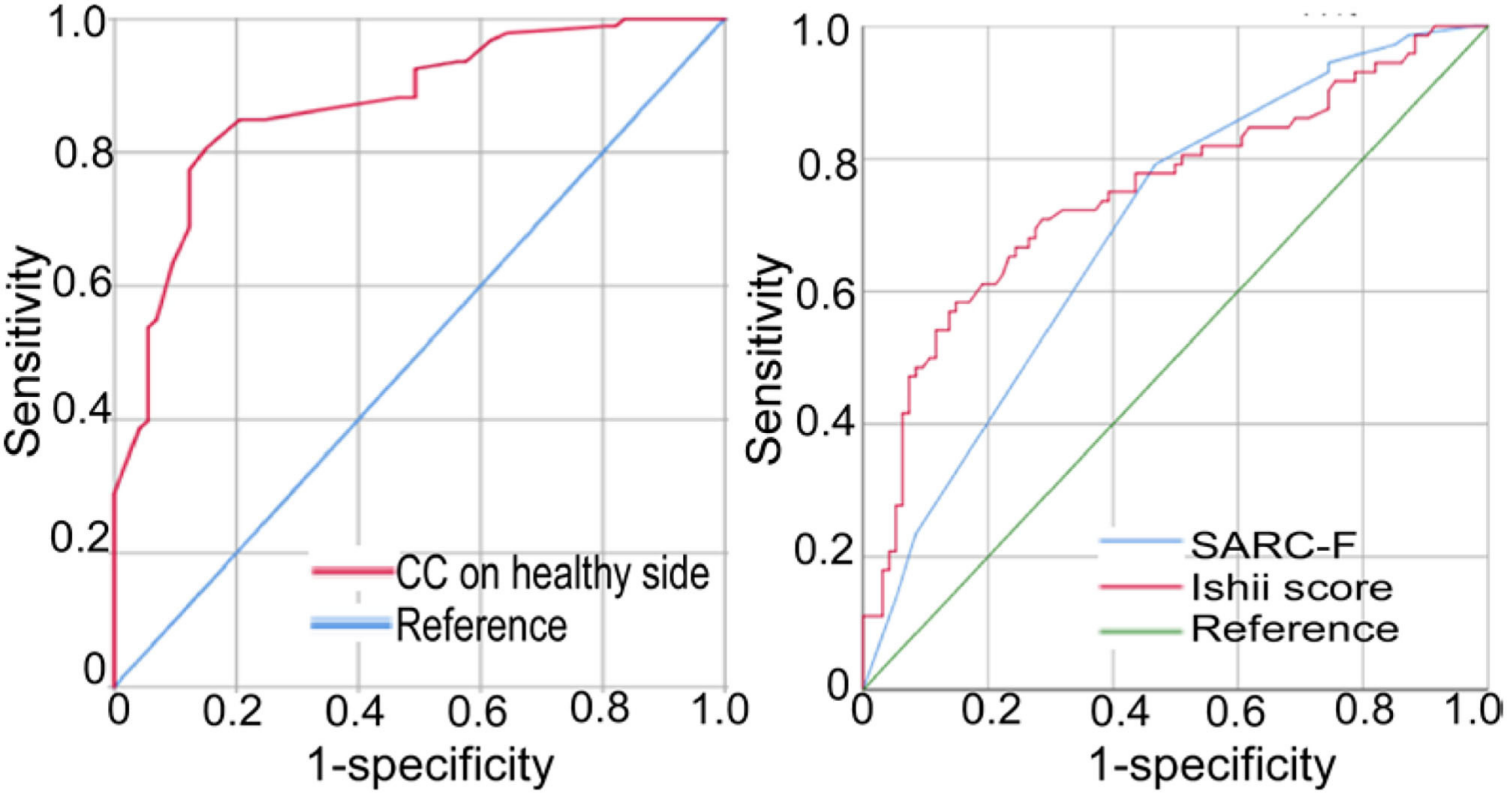
Receiver operating characteristic (ROC) curve analysis was shown of the calf circumference (CC) on the healthy side, the SARC-F questionnaire, and Ishii's score for the diagnosis of stroke-related sarcopenia (SRS) in patients with subacute or chronic stroke.

In ROC curve analysis, the left and right CC measurements had similar effects, with a cutoff value of 31.3 cm, sensitivity of 0.791, Youden index of 0.294, and AUC of 0.887 on the left side and a cutoff value of 31.7, sensitivity of 0.758, specificity of 0.9, Youden index of 0.29, and AUC of 0.89 on the right side (Table 4, Figure 2). The Ishii's score had a cutoff value of 118, sensitivity of 0.901, specificity of 0.36, Youden index of 0.55, and AUC of 0.78. The SARC-F questionnaire had a cutoff value of 5, sensitivity of 0.947, specificity of 0.4, Youden index of 0.41, and AUC of 0.731.

**Table 4:** ROC curve analysis of risk factors for sarcopenia.

**Variables**	**Cutoff**	**Sensitivity**	**Specificity**	**Youden index**	**AUC**
Left CC, cm	31.3	0.791	0.869	0.294	0.887
Right CC, cm	31.7	0.758	0.9	0.29	0.89
Non-paralyzed CC, cm	31.3	0.802	0.859	0.33	0.917
Left upper limb, cm	30.1	0.516	0.95	0.249	0.807
Right upper limb, cm	30.8	0.54	0.94	0.263	0.837
SARC-F	7	0.792	0.54	0.232	0.774
Ishii's score	149	0.58	0.85	0.269	0.827

*ROC, receiver operating characteristic; CC, calf circumference*.

## Discussion

In this study that investigates the accuracy of sarcopenia diagnosis in patients with stroke by CC measurement, SARC-F questionnaire, and Ishii's score in comparison with the 2019 AWGS sarcopenia diagnostic criteria, it was found that the sensitivity, specificity, Youden index, likelihood ratio, predictive value, AUC, and Kappa coefficient of CC measurement were the best in screening SRS compared with those of the SARC-F questionnaire and Ishii's score and could be used in self-screening of sarcopenia. In patients with stroke, the cutoff value of CC for sarcopenia is < 31 cm in men and 30 cm in women, with an AUC of 0.85.

To the best of our knowledge, our study is the first to evaluate the accuracy of CC measurement, the SARC-F questionnaire, and Ishii's score for screening sarcopenia in patients with stroke. Early screening with the CC measurement helps to easily identify a high number of patients with stroke at risk of sarcopenia without resorting to some special equipment. CC measurement has the optimal performance compared to other screening tools, with the sensitivity and specificity of 81.8 and 90.1% in the whole cohort, 78.9 and 95.7% in male patients, and 84.2 and 81.8% in female patients, respectively.

Different societies, mainly including the EWGSOP, IWGS, and AWGS, have different sarcopenia diagnosis criteria. The first measurement parameter was the gait speed in both EWGSOP and IWGS, but the diagnosis criteria were different, with ≤0.8 m/s in EWGSOP and <1.0 m/s in IWGS for sarcopenia diagnosis. The diagnosis criteria of ASMI were ≤7.23 kg/m^2^ in men and ≤5.67 kg/m^2^ in women for IWGS but ≤ 7.0 kg/m^2^ in men and ≤ 6.0 kg/m^2^ in women for EWGSOP. IWGS did not refer to grip, but the second measurement step in EWGSOP was grip followed by measurement of muscle mass. AWGS measured only muscle mass and handgrip strength without the measurement of gait speed, and sarcopenia was diagnosed when both the measurements were less than the normal values.

At present, there are no unanimous consensuses on the diagnostic criteria of SRS, but some evaluation criteria have been widely recognized, such as the diagnostic criteria established by EWGSOP, AWGS, and IWGS, and the National Institutes of Health (FNIH) sarcopenia program is currently the gold standard for sarcopenia diagnosis. Since the gold standard diagnosis criteria require professional instruments and personnel, the wide application is restricted. In recent years, some new screening tools have been present for primary sarcopenia, such as the CC measurement, SARC-F questionnaire, and Ishii score, which have the advantages of timing saving, low cost, and convenience. These tools can be used to detect sarcopenia at the early stage of the disease, thus facilitating timely intervention to improve the quality of life for the patients. Our study confirmed the optimal performance of CC in screening SRS, with the optimal positive and negative likelihood ratios and positive and negative predictive values for diagnosing SRS.

The CC measurement had a Kappa coefficient of 0.72 in the total population, 0.74 in men, and 0.66 in women. The Kappa coefficient was 0.34 for the SARC-F questionnaire in the whole cohort and was 0.4 in the total population, 0.5 in male and 0.40 in female patients for Ishii's score. The high values in the Kappa coefficient of CC measurement indicate a high degree of consistency of CC screening with the AWGS diagnostic criteria, thus worthy of promoting and applying in patients with subacute and chronic stroke.

The AUC was used to compare the overall diagnostic accuracy of three screening tools. The AUC was 0.85 in the total cohort of patients, 0.83 in men, and 0.80 in women for the CC measurement, 0.78 in all patients, 0.8 in men, and 0.77 in women for Ishii's score, and 0.731 in all patients for the SARC-F questionnaire. The best performance was proved to be in the CC measurement rather than in Ishii's score or the SARC-F questionnaire. The CC measurement had the greatest Youden index (0.72 in all patients, 0.66 in men, and 0.62 in women), and based on this, the cutoff value for the CC measurement was chosen as 31 cm for male patients and 30 cm for female patients with sarcopenia. CC had been proved to be positively correlated with ASMI, the diagnostic index of sarcopenia (29, 30). CC had been confirmed as the recommended index for sarcopenia screening evaluation after investigating various physical indicators of the elderly including muscle mass, strength, physical performance, handgrip strength, and walking speed (31). The AWGS 2019 screening and diagnostic criteria for sarcopenia recommended CC < 34 cm in men and CC <33 cm in women for sarcopenia diagnosis based on the CC measurement data of 526 Japanese men over the age of 40 years in comparison with dual-energy X-ray absorption (DXA) (21). In our study that investigates patients with SRS, the CC standard for sarcopenia diagnosis was found to be 31 cm (sensitivity of 78.9% and specificity of 95.7%) in men and 30 cm (sensitivity of 84.2% and specificity of 81.8%) in women. Whether these standards were good for application in a large area still warranted further in-depth investigation.

The CC test, as an easy and quick screening method for SRS, is based on the correlation between CC and human muscle mass. The CC screening test was used for the first time in a Japanese community of older adults with primary sarcopenia as reported in a prospective cohort study to compare the efficiency of CC and SARC-F for diagnosis of sarcopenia (32). The risk of sarcopenia had been proved to be significantly higher in the “ < 34 cm in male and 33 cm in female” groups than in the “equal to and larger than 34 cm in male and 33 cm in female” groups, confirming the effectiveness of the CC screening method (33). The CC screen test had also been used to screen for frailty in the elderly and muscular dystrophy in patients with chronic diseases such as coronary obstructive pulmonary disease (COPD) and heart failure (34–36). By evaluating the accuracy of different approaches for screening SRS, our study found that CC measurement was a simple, rapid, and effective sarcopenia screening method for patients with stroke, with the most suitable cutoff value being set for Chinese patients. In our study that compares the effects of CC measurement, SARC-F questionnaire, and Ishii's score on accurate evaluation of sarcopenia in patients with stroke, only the CC measurement achieved the highest consistency with the reference standard, without the involvement of tedious testing and expensive equipment such as CT and MRI scanners, ultrasound, BIA, and DXA, with 86% of SRS being detectable by CC screening. SRS has been considered a rare event and largely ignored by most doctors. Up to date, there is no reference standard for diagnosis of SRS in the guidelines and consensus on stroke rehabilitation or in the consensus on diagnosis and treatment of sarcopenia. The Asian sarcopenia working group proposed screening and diagnostic criteria in 2019 (18). In our study, the CC cutoff value for SRS diagnosis was found at <30 cm for female and <31 cm for male patients with stroke, with higher specificity and sensitivity. Moreover, the positive and negative predictive values, positive and negative likelihood ratios, and accuracy rate had also been investigated in our study, whereas these parameters were not explored by the Asian sarcopenia working group when proposing the screening and diagnostic criteria of sarcopenia in 2019 (18). In our study, the CC measurement was found to have a higher sensitivity, specificity, positive and negative predictive values, positive likelihood ratio, AUC, and Kappa coefficient but a lower negative likelihood ratio. The Kappa coefficient was 0.7, indicating high consistency. The likelihood ratio refers to the ratio between the probability of a patient having a certain test result (positive or negative) and the probability of a non-patient having the corresponding result. The positive likelihood ratio refers to the number of times the probability of a patient having a positive test result than that of a negative result. The greater the value, the better the screening value of the test. The negative likelihood ratio means *vice versa*. The predictive value refers to the probability of correct judgment of the test results. The greater the value, the better the outcome. A positive predictive value refers to the possibility of a person with positive results suffering from the disease, whereas a negative predictive value refers to the possibility that the person with a negative test outcome will not suffer from the disease. To ensure the authenticity and reliability of the research data, before the start of the test, the relevant calculation standards and diagnostic standards were unified, the relevant instruments were calibrated, and two researchers were instructed by professionals to correctly use the instruments and standardize the operation process. During the experiment, each task was recorded and instructed by the same researcher using the same instructions.

Our study identified that the higher sensitivity for SRS is a major limitation. The sensitivity of rapid diagnosis may improve the effect of rehabilitation on SRS. The use of the CC measurement could increase the likelihood of accurately diagnosing patients with a high risk of sarcopenia so as to improve muscle atrophy caused by malnutrition, inactivity, and denervation (37). The CC screening approach for SRS may be able to replace the BIA method (37), grip strength, and walking speed as the initial screening test because this approach was free, quick, consistent with the standard method of diagnosis, and sensitive to the diagnosis of SRS, especially for patients with stroke with hemiplegia, in a coma, or who were not willing to undergo CT and MRI scans, BIA, and DXA (37–39). If the CC screening was preliminarily positive, confirmatory testing and linkage to care with increased nutrition and exercise would be suggested. If the CC result was negative, an additional examination such as grip strength, walking speed, imaging scanning, DXA, or BIA would be used to confirm the diagnosis (39).

In stratification analysis of patients with ischemic or hemorrhagic stroke, no significant (*p* > 0.05) differences were found in the SARC-F scores, male number, left mid-arm circumference, right CC, left grip strength, and incidence of SARS between ischemic and hemorrhagic strokes. Patients with ischemic stroke were significantly (*p* = 0.001) older than those with hemorrhagic stroke, and the right handgrip strength was significantly (*p* = 0.001) greater in patients with ischemic stroke than those with hemorrhagic stroke. The incidence of SRS was insignificantly (*p* > 0.05) lower in patients with ischemic stroke. In our study, the ischemic stroke was presented in 189 (51.2%) patients and hemorrhagic in 175 (48.8%). Because patients with hemorrhagic stroke usually recovered very quickly, more patients with hemorrhagic stroke were hospitalized in our department for rehabilitation. This is why patients with ischemic and hemorrhagic strokes were similar in percentage.

This study had some limitations, including the one-center design, a small cohort of patients, only Chinese patients enrolled, and the lack of patients with acute stroke, which may all affect the outcome and the generalization of the study. Moreover, our study used a wide interval of stroke duration (2 weeks to 6 months), which may also produce some heterogeneities in the outcome of the study. Future studies will have to resolve all these issues for better outcomes.

## Conclusion

Based on the AWGS 2019 diagnostic criteria, calf circumference has the optimal performance in the sensitivity, specificity, Youden index, likelihood ratio, predictive value, AUC, and Kappa coefficient in screening stroke-related sarcopenia compared with the SARC-F questionnaire and Ishii's score. In patients with stroke, the cutoff value of calf circumference for sarcopenia is < 31 cm in men and 30 cm in women, with an AUC of 0.85. Further research is needed to confirm the diagnostic efficacy of these screening tools for sarcopenia in patients with chronic and acute strokes.

## Data Availability Statement

The raw data supporting the conclusions of this article will be made available by the authors, without undue reservation.

## Ethics Statement

The studies involving human participants were reviewed and approved by the Ethics Committee of the Second Hospital of Kunming Medical University. The patients/participants provided their written informed consent to participate in this study.

## Author Contributions

LY: study design. RY, CY, and B-LG: data collection. RY and B-LG: data analysis. RY: writing of the original version and supervision. B-LG: revision. All authors contributed to the article and approved the submitted version.

## Conflict of Interest

The authors declare that the research was conducted in the absence of any commercial or financial relationships that could be construed as a potential conflict of interest.

## Publisher's Note

All claims expressed in this article are solely those of the authors and do not necessarily represent those of their affiliated organizations, or those of the publisher, the editors and the reviewers. Any product that may be evaluated in this article, or claim that may be made by its manufacturer, is not guaranteed or endorsed by the publisher.
